# A Temperature Sensitive Variant of p53 Drives p53-Dependent MicroRNA Expression without Evidence of Widespread Post-Transcriptional Gene Silencing

**DOI:** 10.1371/journal.pone.0148529

**Published:** 2016-02-03

**Authors:** Miguel A. Cabrita, Erin J. Vanzyl, Jeff D. Hamill, Elysia Pan, Kristen A. Marcellus, Victoria J. Tolls, Rhea C. Alonzi, Alyssa Pastic, Teeghan M. E. Rambo, Hadil Sayed, Bruce C. McKay

**Affiliations:** 1 Centre for Cancer Therapeutics, Ottawa Hospital Research Institute, Ottawa ON; 2 Department of Biology, Carleton University, Ottawa, ON; 3 Institute for Biochemistry, Carleton University, Ottawa, ON; French National Center for Scientific Research - Institut de biologie moléculaire et cellulaire, FRANCE

## Abstract

The p53 tumour suppressor is a transcription factor that can regulate the expression of numerous genes including many encoding proteins and microRNAs (miRNAs). The predominant outcomes of a typical p53 response are the initiation of apoptotic cascades and the activation of cell cycle checkpoints. HT29-tsp53 cells express a temperature sensitive variant of p53 and in the absence of exogenous DNA damage, these cells preferentially undergo G_1_ phase cell cycle arrest at the permissive temperature that correlates with increased expression of the cyclin-dependent kinase inhibitor p21^WAF1^. Recent evidence also suggests that a variety of miRNAs can induce G_1_ arrest by inhibiting the expression of proteins like CDK4 and CDK6. Here we used oligonucleotide microarrays to identify p53-regulated miRNAs that are induced in these cells undergoing G_1_ arrest. At the permissive temperature, the expression of several miRNAs was increased through a combination of either transcriptional or post-transcriptional regulation. In particular, miR-34a-5p, miR-143-3p and miR-145-5p were strongly induced and they reached levels comparable to that of reference miRNAs (miR-191 and miR-103). Importantly, miR-34a-5p and miR-145-5p are known to silence the Cdk4 and/or Cdk6 G_1_ cyclin-dependent kinases (cdks). Surprisingly, there was no p53-dependent decrease in the expression of either of these G_1_ cdks. To search for other potential targets of p53-regulated miRNAs, p53-downregulated mRNAs were identified through parallel microarray analysis of mRNA expression. Once again, there was no clear effect of p53 on the repression of mRNAs under these conditions despite a remarkable increase in p53-induced mRNA expression. Therefore, despite a strong p53 transcriptional response, there was no clear evidence that p53-responsive miRNA contributed to gene silencing. Taken together, the changes in cell cycle distribution in this cell line at the permissive temperature is likely attributable to transcriptional upregulation of the CDKN1A mRNA and p21^WAF1^ protein and not to the down regulation of CDK4 or CDK6 by p53-regulated miRNAs.

## Introduction

Mutations in the p53 tumor suppressor are among the most common genetic alterations in cancer [1, 2]. The p53 protein is best known as a DNA damage-inducible sequence-specific transcription factor [3]. The protein binds to consensus sequence elements in promoters, introns and/or enhancer regions and increases the expression of many genes [4, 5]. Downstream targets of p53 include a variety of genes encoding proteins that can inhibit cell cycle progression, inhibit angiogenesis, increase DNA repair capacity and/or induce apoptosis [3, 6]. The p53 protein can function as a transcriptional repressor of a distinct subset of genes, as well and this may occur through more than one mechanism [7–9]. Collectively, these distinct processes protect cells from neoplastic transformation.

In addition to mRNAs, the p53 protein positively regulates the expression of microRNAs (miRNAs), short evolutionarily conserved RNAs that play critical roles in post-transcriptional and translational silencing of gene expression [10–16]. The mature double stranded miRNA duplexes are generated through sequential processing of hairpin structures present initially in primary miRNAs (pri-miRNAs) in a Drosha- and then Dicer- dependent manner [17, 18]. Individual stands of miRNA duplexes bind in a sequence-directed manner to miRNA recognition elements (MREs) in the 3’UTRs of mRNAs [19, 20]. This interaction can direct the transcript for deadenylation-dependent decay and/or translational inhibition [12, 21].

It has been reported that, p53 can positively regulate miRNA expression through 2 distinct mechanisms. First, p53 functions as a transcriptional activator of specific pri-miRNA genes like the MIR34A gene [13, 15, 16, 22]. In addition, p53 can stimulate the processing of specific miRNAs without a corresponding increase in the synthesis of the pri-miRNA (i.e. miR-143-3p and miR-145-5p) [23, 24]. The p53-dependent induction of specific miRNAs is thought to be important for both p53-mediated cell cycle arrest and p53-dependent apoptosis [13, 15, 16, 25]. In this way, p53 has the potential to indirectly inhibit the expression of many proteins and this, in turn, is thought to contribute to p53-mediated tumour suppression.

There are a variety of conditional expression systems that can be used to study p53 activity in the absence of exogenous DNA damage [26–28]. The V135A variant of murine p53 is temperature-sensitive for nuclear import so it provides a way of modulating p53 transcriptional activity [29, 30]. At the restrictive temperature, this variant of p53 is primarily cytoplasmic where it cannot function as a sequence-specific transcription factor but at the permissive temperature, p53 rapidly enters the nucleus where it stimulates p53-dependent gene expression [27–32].

We have used a stable transgenic cell line derived from HT29 colon cancer cells that express the V135A variant of p53 (HT29-tsp53) to study the p53 transcriptional, apoptotic and cell cycle checkpoint responses [28, 31, 32]. HT29-tsp53 cells undergo a rapid G_1_ cell cycle arrest at the permissive temperature that correlates with increased expression of the cyclin-dependent kinase inhibitor p21^WAF1^ without widespread apoptosis [28, 31, 32]. However, p53 regulates miRNAs that target mRNAs encoding cell cycle regulatory proteins like Cdk4 and Cdk6 [33–35]. The contribution of p53-regulated miRNAs to this cellular response was unknown; therefore, we sought to identify differentially expressed miRNAs in these cells using oligonucleotide microarrays. A subset of miRNAs were increased at the permissive temperature through both transcriptional and post-transcriptional mechanisms. However, there were comparatively few down regulated mRNAs at the permissive temperature and the p53-induced miRNAs didn’t appear to have a marked effect on the expression of downregulated transcripts or known targets of these miRNAs, including Cdk4 and Cdk6. Our results suggest that transactivation of p53-dependent gene expression had a greater impact than miRNA-mediated gene silencing in these cells that undergo cell cycle arrest.

## Materials and Methods

### Cell culture

All cells were grown at 37°C (restrictive temperature) in Dulbecco’s modified Eagle’s medium (DMEM) supplemented with 10% fetal bovine serum (FBS) in humidified incubator with 5% CO2. HT29 cells are human colon cancer cells homozygous for an R273H mutation that renders the endogenous p53 protein transcriptionally inactive [36]. HT29-tsp53 cells were derived from this tumour cell line following transfection of cDNA encoding a temperature sensitive variant of murine p53 (V135A) [37].

### Microarray analysis

HT29-tsp53 cells were switched to the permissive temperature (32°C) for 16 h and samples were collected for comparison to a control sample maintained at the restrictive temperature (37°C). RNA was collected for miRNA analysis using the Qiagen miRNeasy Mini Kit (Qiagen, Valencia, CA) according to the manufacturer's recommendations. Samples were submitted for analysis at the Stemcore Labs Affymetrix Gene-Chip Facility at the Ottawa Hospital Research Institute (Ottawa, ON) using miRNA 1.0 oligonucleotide microarrays (Affymetrix, Santa Clara, CA) (Gene Expression Omnibus (GEO) accession #GSE76576). Robust Multi-array Average (RMA) and an Empirical Bayesian model [38] were used to identify statistically significant (P < 0.01) differences in miRNA expression (FlexArray 1.4.1, Genome Quebec http://genomequebec.mcgill.ca/FlexArray).

Total RNA was isolated using the RNeasy RNA isolation kit (Qiagen) according to the manufacturer's specifications and these samples were also submitted for analysis at Stemcore Labs with the Human Gene 1.0 ST oligonucleotide microarrays (Affymetrix) (GEO accession #GSE76575). Probesets in the Human Gene 1.0 ST arrays span the entire locus with each probeset representing an individual exon so individual transcripts are represented by multiple probesets that can be readily identified by a common cluster identification number. Significant changes in the expression at individual probesets were identified (P<0.001) using FlexArray 1.4.1 software with the Affymetrix Power Tools software package based on an empirical Bayesian algorithm [38]. Fold change in mRNA expression was determined using expression data for all linked probesets (shared cluster identification number). Analysis of up-regulated but not down-regulated transcripts was reported previously [32].

### Quantitative reverse transcriptase polymerase chain reaction

The Taqman Small RNA Assays (Life Technologies, Burlington, ON) were employed to quantitate relative quantities of miRNAs. First, total RNA (30ng/reaction) was reverse transcribed in multiplex (3–5 miRNA-specific primers/reaction) using Taqman MicroRNA Reverse Transcription kit (Life Technologies, Burlington, ON). These reactions were diluted 1:3 in nuclease-free water. Next, specific Taqman miRNA primers were used along with Taqman Universal PCR Master Mix II to measure the relative amounts of miRNAs on an Applied Biosystems 7500 FAST Real-Time PCR system (Life Technologies, Burlington, ON). Two miRNAs, miRNA 103 and miRNA 191, were used as endogenous controls for normalization as little to no fluctuation in the levels of this pair of miRNAs was observed upon activation of p53. All reactions were performed in triplicate for each miRNA.

Total RNA was isolated as described above. Five micrograms of total RNA was reverse-transcribed using first-strand cDNA synthesis kit (MBI Fermentas, Burlington, ON). Quantitative reverse transcriptase polymerase chain reaction (qRT-PCR) was performed with a Step One Plus thermocycler and with the following Taqman primers sets: Hs00198887_m1, Hs03044953_m1, Hs00355782_m1, Hs00165683_g1, Hs01026371_m1, Hs02758991_g1, Hs00393722_m1, Hs00364284_g1, Hs00969422_m1, Hs01066930_m1, Hs00153408_m1, Hs01126606_m1, Hs00374522_m1 and Hs00323234_m1 (Life Technologies, Burlington, ON).

### Immunoblot analysis

Cells were rinsed twice with 2 ml of PBS (pH 7.4) and then collected by scraping in 600 μL RIPA buffer followed by sonication. Protein concentration was determined using the Bio-Rad protein assay with an xMark Microplate Absorbance Spectrophotometer at a wavelength at 595nm. Equal amounts of protein was loaded per well of NuPAGE 10% Bis-Tris precast polyacrylamide gels. Electrophoresis with NuPAGE MOPS SDS Running buffer was performed at 200 volts for between 45 and 60 min. Proteins were transferred to Hybond-C nitrocellulose (GE Healthcare, Baie d’Urfé, QC) membranes and these blots were stained with 5 mg/ml Ponceau S Red in 2% glacial acetic acid to visualize total transferred proteins. Membranes were blocked for at least 1 hour in 5% non-fat milk in TBST, washed in TBST (4 x 5 min), the primary antibody was suspended in 0.5% non-fat milk and incubated with the membrane for at least 2 hours at room temperature, followed by four 5 min washes in TBST. The membrane was then incubated in either goat-anti-mouse or goat-anti-rabbit horse radish peroxidase conjugated secondary antibody in TBST for 1 hour followed by four 5 minute washes in TBST and 5 min in 1 mL of SuperSignal West Pico Chemiluminescent Substrate (Thermo Fisher Scientific) before imaging with a Fusion FX5 gel documentation system (Vilber Lourmat, France). The membranes were then stripped for 15 minutes in Restore PLUS Western Blot Stripper Buffer (Fisher Scientific) and probed sequentially with additional primary antibodies, as described above. The primary antibodies included β-actin (Sigma-Aldrich), p21 (Calbiochem), Cdk4 (Santa Cruz), Cdk6 (Santa Cruz) K-Ras (Santa Cruz), Lgr5 (Thermo Scientific) and c-Myc (Santa Cruz)

### Flow cytometry

The incorporation of 5-bromo-2’-deoxyuridine (BrdU) was used to identify actively replicating S-phase cells by two parameter flow cytometry. Cells were incubated with BrdU (30 μM; Sigma-Aldrich Canada Ltd.) for 30 min immediately prior to collection in order to label nascent DNA. Cells were collected and fixed at -20°C in 70% ethanol for a minimum of 1 h. Detection of BrdU incorporated into single- stranded DNA was performed using a primary anti-BrdU antibody (1:100; Phar- Mingen) and secondary anti-mouse FITC-conjugated antibody (1:15; Sigma-Aldrich Canada Ltd.), as described previously [39]. DNA was stained with propidium iodide (30 μM in PBS) and then samples were analyzed by fluorescence-activated cell sorting using a Becton Dickinson Accuri C6 benchtop flow cytometer and FCS Express 3 software (DeNovo Software). One parameter flow cytometry experiments were performed similarly except that cells were not incubated in BrdU and the fixed cells were simply stained in 30 μM propidium iodide in PBS. Cell cycle distribution in one parameter flow cytometry experiments was determined based on DNA content using Modfit 4.1 (Verity Software House).

## Results

### A subset of p53-responsive miRNAs accumulate to high levels in HT29-tsp53 cells

**Fig 1:**
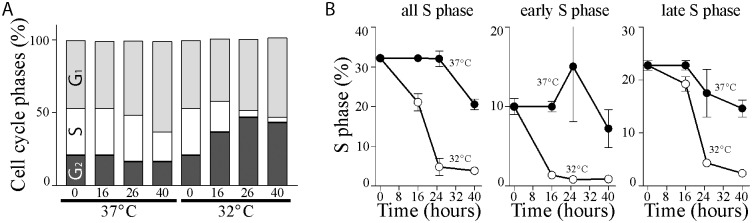
G_1_ and G_2_ arrest are induced at the permissive temperature in HT29tsp53 cells. (A) Two parameter flow cytometric analysis was used to determine cell cycle distribution as a function of DNA content (PI stain) and DNA replication (BrdU incorporation). Each column represents the percentage of cells under each condition in each phase of the cell cycle. (B) The S phase population in (A) was further separated into early and late S phase based on DNA content and plotted as a function of time. Each value in (A) and (B) were determined from the same experiments and each represents the mean (± SEM) determined from a minimum of 3 independent experiments.

HT29-tsp53 cells express the V135A variant of murine p53 that is temperature-sensitive for nuclear import [29, 30]. We previously reported that HT29-tsp53 cells rapidly undergo a G_1_ cell cycle arrest at the permissive temperature [31, 32]. Here we find that these cells also accumulated with 4C DNA content when maintained at the permissive temperature for 16 hours or more (Fig 1A). This increase in the proportion of cells in G_2_ and/or M phase is associated with loss of the S phase population with little effect on the proportion of cells in G_1_ (Fig 1A). Detailed quantification of early and late S phase populations, determined by DNA content, indicated that the early S phase population was depleted a few hours before the late S phase population (Fig 1B). These changes in cell cycle distribution are consistent with reported roles of p53 in mediating both G_1_ and G_2_ cell cycle arrests [40–42].

**Fig 2:**
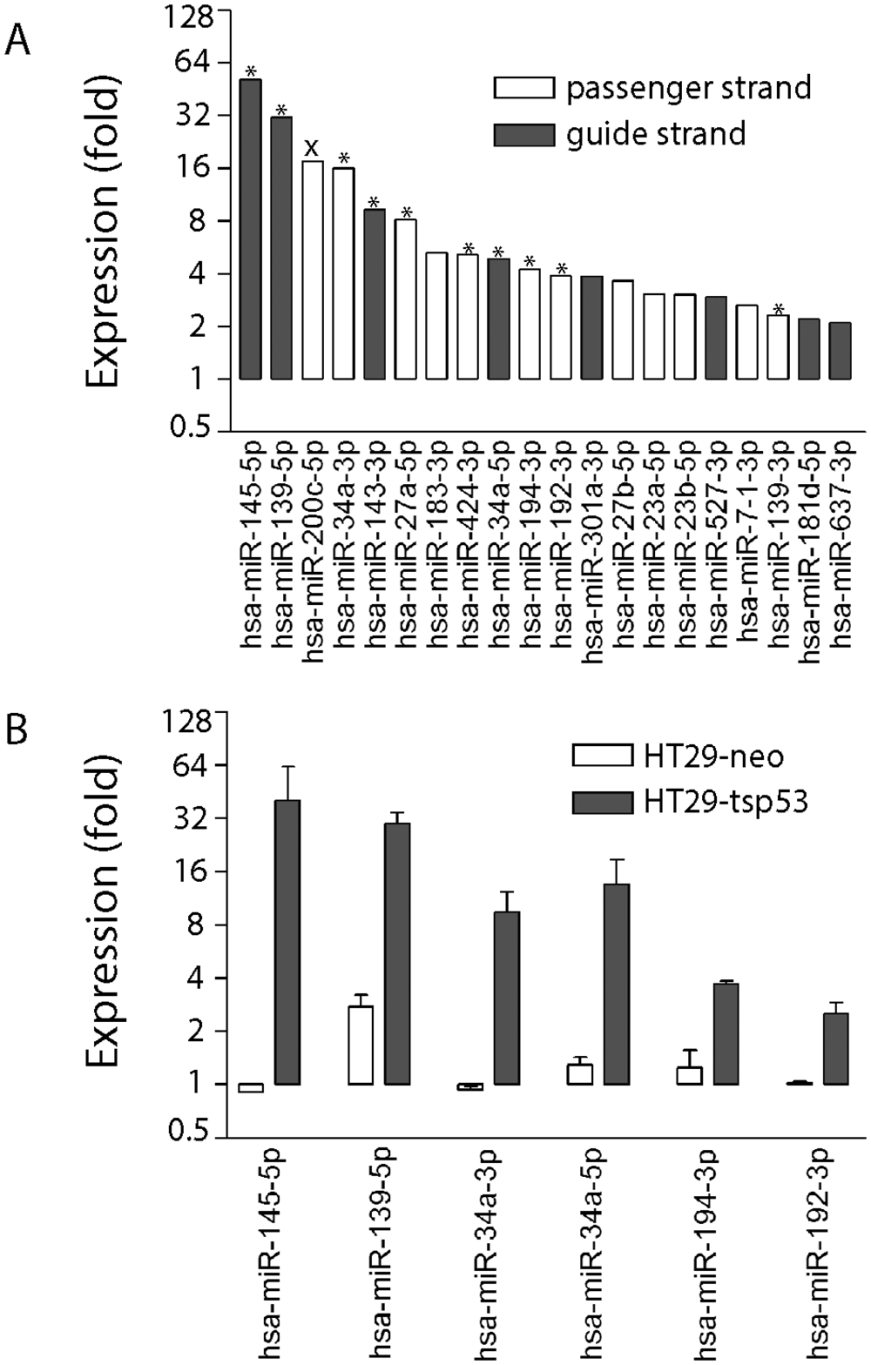
The p53-dependent induction of miRNAs. (A) Small RNAs were collected from HT29-tsp53 cells following incubation for 16 hours at the permissive temperature. RNA samples obtained from 2 independent experiments were labelled and hybridized to oligonucleotide microarrays. Individual miRNAs meeting our statistical cut off (P ≤ 0.01) are presented. Values reflect fold change in expression at the permissive compared to the restrictive temperature. The miRNAs are arranged in order of expression from most highly induced on the left to least well-induced on the right. Open bars denote passenger strand miRNAs while solid bars represent guide strand miRNAs. ‘*’ indicates that the increased expression of the miRNA was confirmed by qRT-PCR. The ‘X’ denotes the only miRNA that we could not confirm by qRT-PCR. (B) qRT-PCR was performed using similar samples derived from vector control (HT29-neo) and HT29-tsp53 cells. Each value in (B) represents the mean (± SEM) determined from a minimum of 3 independent experiments.

Affymetrix oligonucleotide microarrays (Affymetrix miRNA 1.0 oligonucleotide microarrays) were then used to monitor p53-dependent changes in miRNA expression at 16 hours, after the onset of both G_1_ and G_2_ arrests (Fig 2A and 2B). The expression of 21 miRNAs were statistically increased at the permissive temperature compared to the restrictive temperature (P < 0.01) (Fig 2A). The 21 miRNAs were encoded from only 13 pri-miRNAs because most miRNAs were linked to other miRNAs either as part of bicistronic miRNA genes or as opposite strands of the same miRNA duplex (Table 1). The increased expression of 10 of 11 miRNAs examined was confirmed independently using qRT-PCR (Fig 2A). Only miR-200c-5p was identified as a false positive in the microarray analysis. A subset of these miRNAs were also quantified under identical conditions in vector control cells and all of these miRNAs increased in a p53-dependent manner (Fig 2B). Therefore, p53 increases the expression of many miRNAs at the permissive temperature in this cell line. Not surprisingly, many of the miRNAs identified are known targets of p53 [13–16, 24].

**Table 1:** List of miRNAs induced at the permissive temperature grouped by locus.

Locus^a^	miRNA duplex	Guide strand	increased	Passenger strand	increased
MIR27A^b^	miR27a	miR-27a-3p	N	miR-27a-5p	Y*
MIR23A	miR23a	miR-23a-3p	N	miR-23a-5p	Y
MIR34A	miR-34a	miR-34a-5p	Y*	miR-34a-3p	Y*
MIR143HG	miR-143 ^c^	miR-143-3p	Y*	miR-143-5p	N
	miR-145	miR-145-5p	Y*	miR-145-3p	N
MIR183	miR-183	miR-183-5p	N	miR-183-3p	Y
MIR194-2HG	miR-194-2 ^c^	miR-194-2-3p	N	miR-194-2-5p	Y*
	miR-192	miR-192-3p	N	miR-192-5p	Y*
MIR424^b^	MIR424	miR-424-5p	N	miR-424-3p	Y*
MIR503HG	MIR503HG		N		NP
C9ORF3^d^	miR-27b	miR-27b-3p	N	miR-27b-5p	Y
	miR-23b	miR-23b-3p	N	miR-23b-5p	Y
DAPK3 ^d^	miR-637	miR-637-3p	Y	miR-637-5p	NP
HNRNPK ^d^	miR-7-1	miR-7-1-5p	N	miR-7-1-3p	Y
NANOS3 ^d^	miR181D	miR-181d-5p	Y	miR-181d-3p	NP
	miR181C	miR-181c-5p	N	miR-181c-3p	N
PDE2A ^d^	miR-139	miR-139-5p	Y*	miR-139-3p	Y*
SKA2 ^d^	miR-301a	miR-301a-3p	Y	miR-301a-5p	NP
	miR-454	miR-454-3p	N	miR-454-5p	N

^a^ Official gene symbol.

^b^ These two loci are within 500 nucleotides but there is no annotated transcript spanning them.

^c^ The indicated locus encodes a bicistronic pri-miRNA.

^d^ The indicated miRNA(s) is/are expressed from introns in the indicated gene.

* indicates the increased expression of the indicated miRNA was confirmed by qRT-PCR.

To gauge the level of these miRNAs compared to known functional miRNAs in colon cancer cells, we compared the expression of several miRNAs relative to specific reference miRNAs (miR-191 and miR-103). These specific reference miRNAs were selected because they were reported to be stably expressed and functional across multiple cell types and tissues including colon [43–45]. These miRNAs provide a comparator to estimate physiological levels of miRNAs.

**Fig 3:**
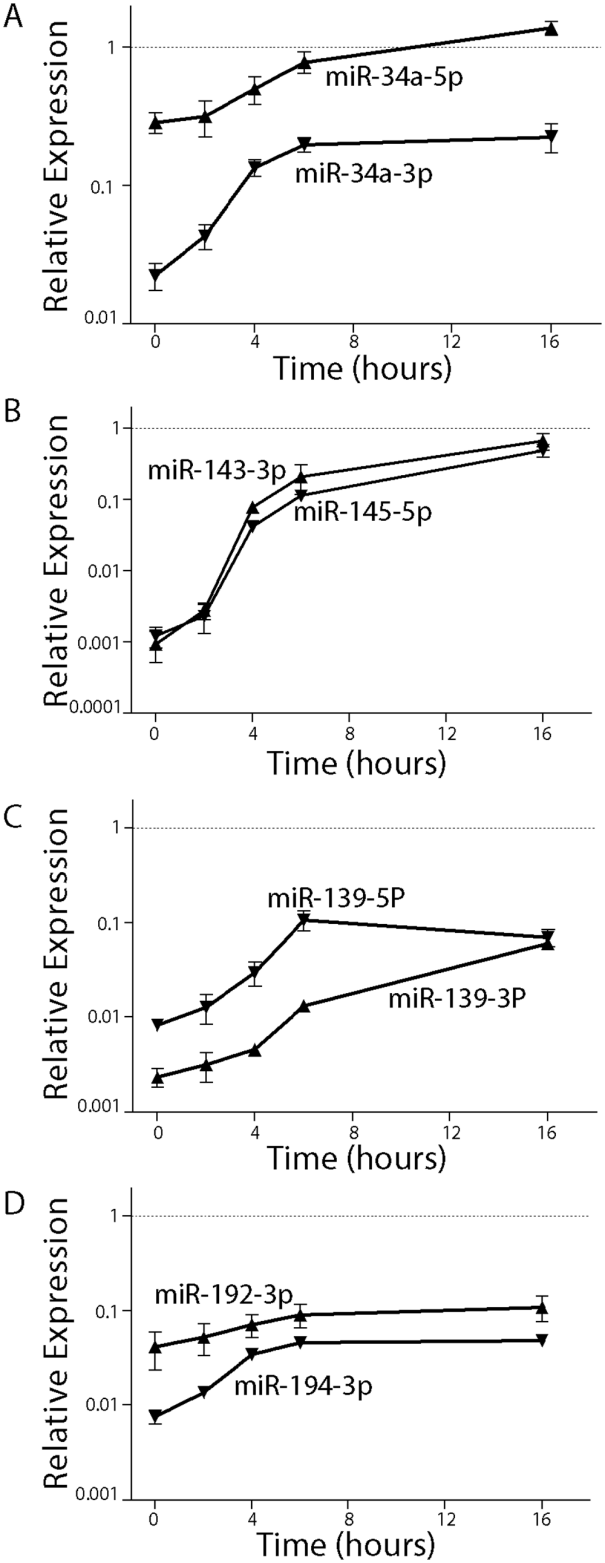
Comparison p53-induced miRNAs to reference RNA levels. CT values were used to compare the indicated miRNA (A-D) to miR-103 and miR-191 expression. Samples were collected following incubation at the permissive temperature for the indicated time. Each point represents the mean (± SEM) determined from a minimum of 3 independent experiments.

For all miRNAs tested, the basal level of expression at 0 hours was well below the level of the reference miRNAs (for example, Fig 3A–3D). Only miRNA-34a-5p levels were within 10 fold of the reference miRNAs, initially (Fig 3A). The level of each p53-regulated miRNA increased but only miR-34a-5p, miR-143-3p and miR-145-5p reached a level comparable to that of the reference miRNAs (Fig 3A and 3B). Neither the levels of miR-139-5p nor the levels of any passenger strand miRNAs (miR-34-3p, miR-192-3p, miR-194-3p and miR-139-3p) reached this level. Therefore, miR-34a-5p, miR-143-3p and miR-145-5p were considered most likely to have physiologically relevant effects on target gene expression.

### Prolonged expression of p53-regulated miRNAs

**Fig 4:**
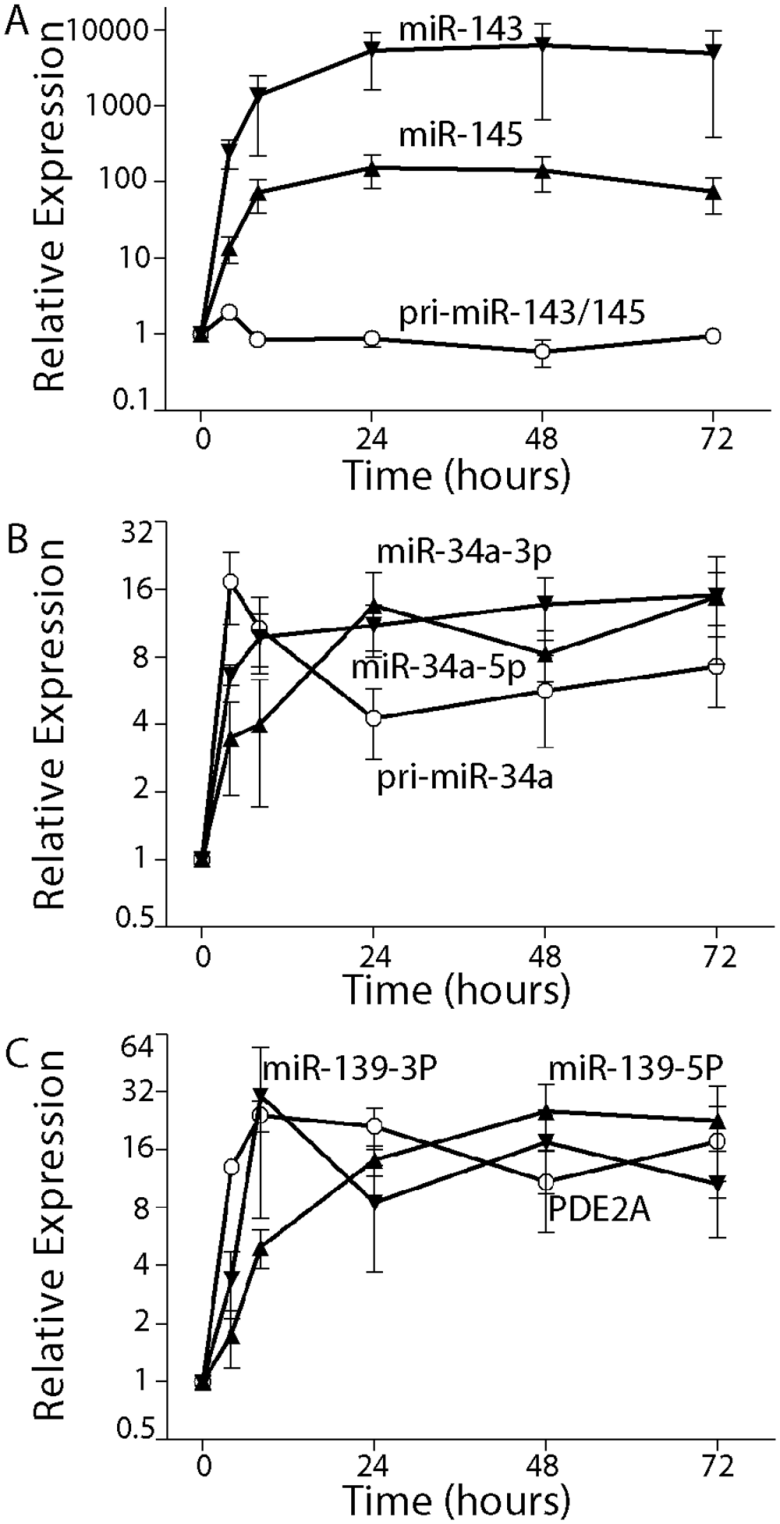
The induction of miRNAs and their respective pri-miRNA. The fold increase in RNA expression for the indicated miRNAs (closed symbols) and their respective pri-miRNAs (open symbols) is plotted as a function of time. Each point represents the mean (± SEM) determined from a minimum of 3 independent experiments.

Given that HT29-tsp53 preferentially undergo prolonged cell cycle arrests *in lieu* of apoptosis [28, 31, 32], we were able to monitor the expression of several of the highly induced p53-regulated miRNAs over a 3 day period (Fig 4A–4C). The expression of these miRNAs remained elevated even after 72 hours at the permissive temperature. We also used qRT-PCR to measure the corresponding pri-miRNAs under identical conditions to determine whether each was regulated transcriptionally or post-transcriptionally. The pri-miRNAs exhibited distinct patterns at the permissive temperature.

First, there was no increase in the expression of the bi-cistronic pri-miRNA encoding miR-143-3p and miR-145-5p, despite the sustained expression of the processed miRNAs (Fig 4A). Thus, increased miR-143-3P and miR-145-5p is most consistent with the previously reported post-transcriptional mechanism [24, 46]. This was deduced from the fact that for a small increase in pri-miRNA (1.3 fold) to yield very large increases in the mature product (50 to 70 fold), the rate of conversion of nascent pri-miRNA would have to be higher that the processing rate under the basal condition. Importantly, our results indicate that the V135A variant of p53 retains the ability to stimulate post-transcriptional processing of these pri-miRNAs and that this post-transcriptional activity of p53 is regulated in the same temperature sensitive manner.

Second, the pri-miR-34a transcript encoding miR-34a-5p and miR-34a-3p (i.e. passenger strand of miR-34a-5p) accumulated strongly within 4 hours consistent with a rapid transcriptional response [13, 15, 16]. The pri-miRNA levels subsequently declined somewhat but the mature miRNAs remained elevated throughout the course of the experiment (Fig 4B). Therefore, the miR-34a family miRNAs increased through a rapid p53-dependent transcriptional response, as previously reported [13–16].

Third, miR-139-5p and miR139-3p (passenger strand of miR-139-5P) increased rapidly, as well (Fig 4C). However, these miRNAs are expressed from an intron in the PDE2A gene and increased expression of the miRNAs was associated with increased expression of the host gene (Fig 4C). Therefore, the mir-139 family miRNAs increased through a rapid p53-dependent transcriptional response [47].This is in apparent contrast to their reported post-transcriptional regulation in another colon cancer cell line [48]. Taken together, this temperature sensitive variant of p53 controls the expression of several p53-regulated miRNAs through at least 2 distinct mechanisms.

### Expression of known targets of miR-34a-5p, miR-143-3p and miR-145-5p

**Fig 5:**
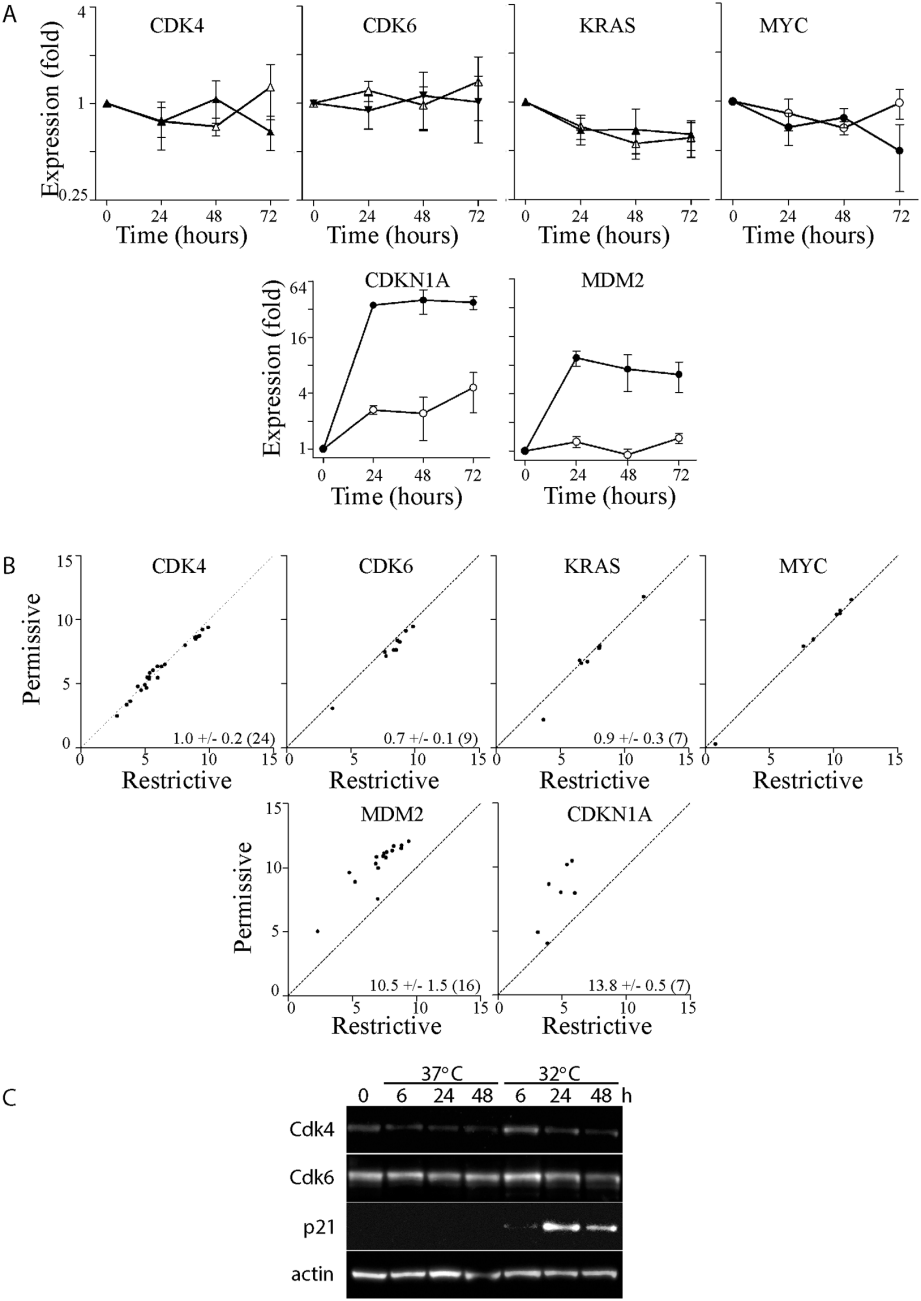
The expression of CDK4 and CDK6 at the permissive temperature. (A) Total RNA was isolated from control (open symbols) and HT29tsp53 (closed symbols) cells at the indicated time and the expression of a variety of mRNAs was determined. Expression in each sample was normalized to the expression of the same transcript in samples collected immediately before the temperature shift. (Each point represents the mean (± SEM) determined from a minimum of 3 independent experiments. (B) Signal intensity of individual probesets from microarray analysis at the restrictive temperature (X axis) is compared to the signal intensity at the permissive temperature (y axis). The mean fold change (± SEM and n) for each transcript is inset in the corresponding panel. (C) Immunoblot analysis of the indicated protein derived from whole cell lysates collected at the indicated time at the permissive temperature. Similar results were obtained in 3 independent experiments. In A, B and C, CDKN1A (p21 ^**WAF**^) and/or MDM2 serve as positive controls for the activation of the p53 transcriptional response.

We used miRTarBase, a database of experimentally validated miRNA-targets [49], to identify known targets of these miRNAs that could be downregulated under our experimental conditions. The miR-34a-5p miRNA reportedly targets MYC, CDK4 and CDK6 mRNAs in human cells [14, 35, 50–52]. KRAS is a reported target of miR-143-3p [53, 54] while miR-145-5p is reported to silence CDK4 and MYC [54, 55]. So MYC and CDK4 are targets of at least 2 p53-responsive miRNAs while KRAS and CDK6 are targeted by at least 1. MYC and KRAS mRNAs decreased slowly over a 3 day period in HT29tsp53 cells, however, a similar decrease in MYC and KRAS expression was also detected in control cells lacking functional p53 and is thus independent of p53 and the p53-dependent induction of miR-34a-5p, miR-143-3p and miR-145-5p (Fig 5A). In addition, CDK4 and CDK6 mRNA did not change significantly despite the induction of our positive control mRNAs: the p53-responsive CDKN1A (encoding the cyclin-dependent kinase inhibitor p21, also known as WAF1 and CIP1) and MDM2 (Fig 5A). Taken together, there was no evidence that p53-dependent increases in miR-34a-5p, miR-143-3p or miR-145-5p negatively regulated the expression of any of these characterized target mRNAs.

We had previously used oligonucleotide Affymetrix Human Gene 1.0 ST oligonucleotide microarrays to identify p53-induced mRNAs under these conditions [32]. These experiments provided exon (probeset) level expression data for mRNAs downregulated at the permissive temperature, as well. Consistent with qRT-PCR analysis, we found that there was no significant decrease in the expression of CDK4, CDK6, KRAS or MYC mRNAs following a 16 hour incubation at the permissive temperature despite the robust induction of our positive controls, MDM2 and CDKN1A mRNAs (Fig 5B). The Cdk4 and Cdk6 proteins play important roles at the restriction point so their inhibition by p53 regulated miRNAs could contribute to G_1_ arrest initiated at the permissive temperature in HT29-tsp53 cells [56, 57]. However, no decrease in Cdk4 or Cdk6 protein expression was detected at the permissive temperature despite the increase in expression of our positive control p21 ^**WAF**^ (Fig 5C). Taken together, p53 appeared to have a greater effect on transactivation of known target mRNAs than on miRNA-mediated destabilization of the known targets of miR-34a-5p, miR-143-3p or miR-145-5p examined.

### Identification of downregulated mRNAs

**Fig 6:**
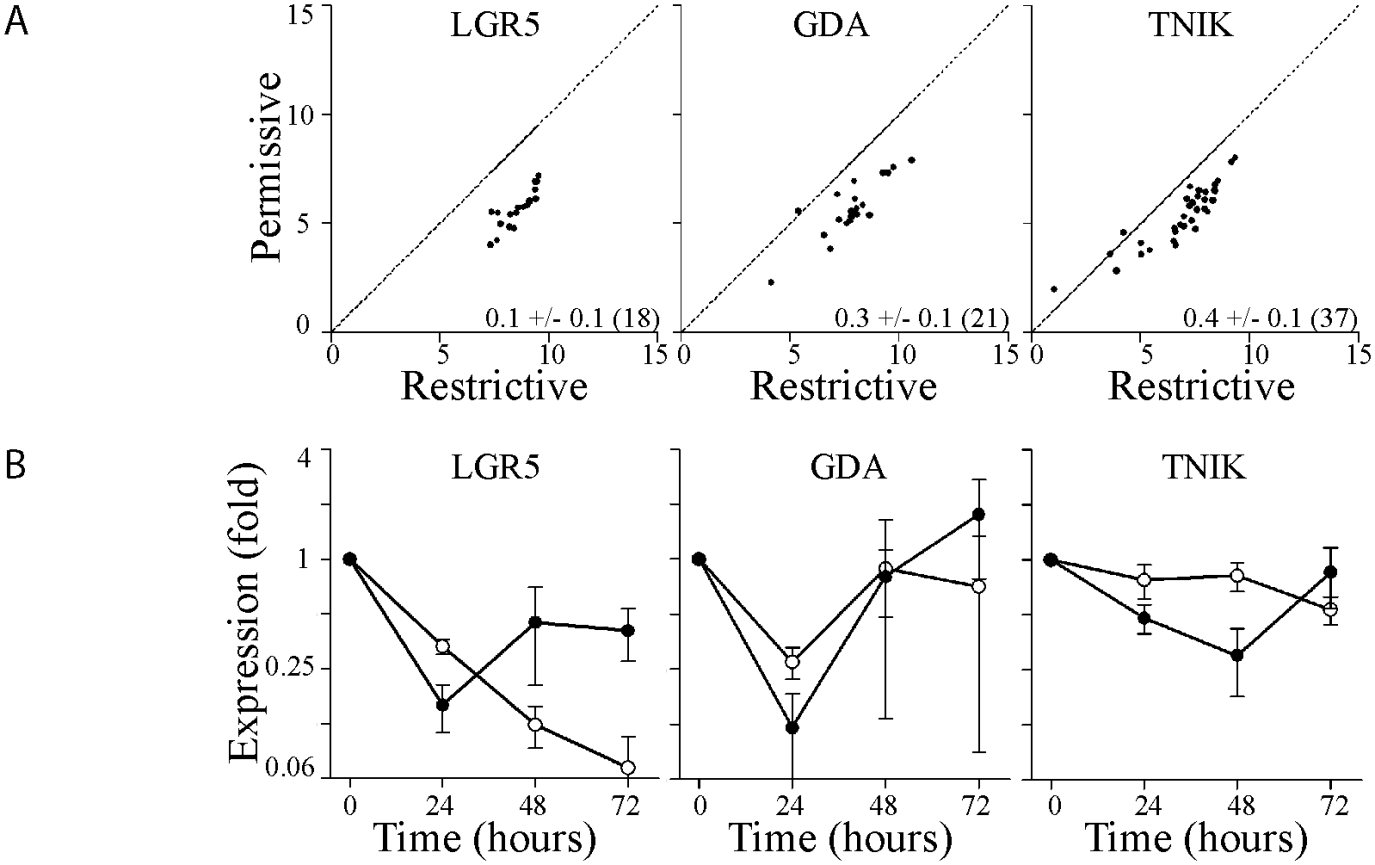
Transcripts downregulated at the permissive temperature were not strictly p53-dependent. (A) Signal intensity of individual probesets from microarray analysis at the restrictive temperature (X axis) is compared to the signal intensity at the permissive temperature (y axis). The mean fold change (± SEM and n) for each transcript is inset in the corresponding panel. RNA samples were collected at 16h following temperature shift. (B) Total RNA was isolated from control (open symbols) and HT29tsp53 (closed symbols) cells at the indicated time and the expression of a variety of mRNAs was determined. Expression in each sample was normalized to the expression of the same transcript in samples collected immediately before the temperature shift. (Each point represents the mean (± SEM) determined from a minimum of 3 independent experiments.

We were somewhat surprised to find that targets of miR-34a-5p, miR-143-3p and miR-145-5p, well supported in the literature, were not detectably silenced in these long term experiments despite the robust induction of miR-34a-5p, miR-143-3p and miR-145-5p. We analyzed our previously published Affymetrix Human Gene 1.0 ST oligonucleotide microarray data [32] obtained under identical conditions to identify mRNAs that were statistically decreased at the permissive temperature (P < 0.001). Using this definition, we identified 183 probe sets representing 145 independent transcripts. Probesets on Human Gene 1.0 ST oligonucleotide microarrays are designed to span single exons so individual genes are represented by multiple probesets that cover the entire mRNA. The similarity between probeset and transcript number suggests that very few mRNAs were in fact down-regulated, the vast majority of changes were at the individual exon level and this is not consistent with miRNA-mediated gene silencing or even p53-dependent transcriptional repression. Nonetheless, 12 transcripts were significantly reduced at 2 or more probe sets and 4 of these mRNAs were reduced more than 2 fold across the entire gene (Table 2). For example, the expression of TNIK, GDA and LGR5 were significantly reduced across the entire length of the gene (Fig 6A). These patterns of mRNA expression were confirmed by qRT-PCR at 24 hours (Fig 6B). However, these decreases in gene expression were not p53-dependent because there was no statistically significant difference between the responses observed in HT29-neo and HT29-tsp53 cells (Fig 6B, P > 0.05 Two-way ANOVA). In contrast, the expression our positive controls, p21^WAF1^ and MDM2 differed significantly between cell lines (P < 0.001, Fig 5A). So once again, there was no evidence that changes in gene expression could be attributed to the p53-dependent changes in the expression of miR-34a-5p, miR-143-3p or miR-145-5p. Our data also suggests that this variant of p53 did not strongly repress transcription of any mRNAs at the permissive temperature. Increased expression of p53-responsive genes was the predominant change in gene expression detected.

**Table 2:** Transcripts reduced at the permissive temperature at a minimum of 2 probe sets.

transcript	Cluster ID #	PROBE SETS (P<0.001)	PROBE SETS ALL	RELATIVE EXPRESSION
LGR5	7957140	11	18	0.14 ± 0.01*
FAM111B	7940147	2	5	0.23 ± 0.03*
GDA	8155802	5	21	0.28 ± 0.05*
TNIK	8092095	3	36	0.42 ± 0.06*
NAPEPLD	8141872	2	9	0.47 ± 0.17
FAM178A	7929858	3	23	0.49 ± 0.09
CLDN2	8169210	2	8	0.51 ± 0.16
NRP1	7932985	2	22	0.66 ± 0.14
C6orf170	8129273	2	35	0.66 ± 0.06
TTC21B	8056426	2	30	0.72 ± 0.07
CCDC7	7926936	2	42	0.83 ± 0.08
TTN	8057056	2	358	1.24± 0.05

* the indicated value is significantly less than 0.5 (P < 0.05, single sample t test).

## Discussion

The p53 protein can induce the expression of miRNAs through transcriptional and post-transcriptional mechanisms [58]. Through our microarray and qRT-PCR analysis, we found that this temperature sensitive variant of p53 appears to be able to increase specific p53-responsive miRNAs through both described mechanisms. We detected transcriptional induction of pri-miRNAs containing the miR-34 and miR-139 hairpins. In contrast, the MIR143HG pri-miRNA was not transcriptionally upregulated despite large fold increases in the expression of both miR-143-3p and miR-145-5p miRNAs. Ultimately, we found that the same common set of p53 responsive miRNAs were increased at the permissive temperature in HT29-tsp53 cells as those frequently reported in the literature in other model systems [13–16, 23, 24, 47]. Therefore, there is incredible congruity in the identification of p53-regulated miRNAs using different cell lines and different models of p53 activation. Among these commonly identified p53-regulated miRNAs, miR-34a-5p miRNA appears to be p53-responsive in all reports [13–16, 23, 24, 47].

Despite the reproducible p53-dependent induction of miR-34a-5p in many independent studies, the identification of *bonafide* target mRNAs has been far more variable. For example, two laboratories originally reported in 2007 that miR-34a-5p was transcriptionally upregulated by p53 and then used microarray analysis to identify downregulated target mRNAs [14, 16]. In one case, transcripts encoding cell cycle proteins (including CDK4) were over-represented among the silenced transcripts and cells underwent a cell cycle arrest [14]. Conversely, transcripts encoding cell cycle regulatory proteins were over-represented among the upregulated transcripts in the other report and cells underwent apoptosis instead [16]. In both publications, the down-regulated mRNAs were enriched for sequences complementary to the miR-34a-5p seed region suggesting that many of the downregulated transcripts were directly targeted by this miRNA [14, 16]. Differences in target mRNAs could reflect cell line specific effects or the fact that these groups used different strategies to express miR-34a-5p. In either case, there must be additional factors that affect the efficiency with which this miRNA inhibits the expression of specific target genes.

In our work, we found no significant decrease in the expression of CDK4, CDK6, MYC or KRAS expression. These mRNAs were previously reported targets of p53-regulated miRNAs induced under our experimental conditions [14, 35, 50–55]. Our oligonucleotide microarray analysis did not identify any clear targets of these miRNAs either. This may reflect the fact that modulation of endogenous miRNA expression generally has modest effects on mRNA and protein expression [12]. Recently, Kozomara and coworkers used reporter genes to study over 30 different endogenous miRNAs in *Drosophila* and they concluded that on average a 10 fold difference in the level of endogenous miRNA expression equated to only a 10% decrease in gene expression [59]. Furthermore, there were specific examples of miRNAs that were expressed at very different levels (orders of magnitude different) but exerted similar levels of gene silencing [59]. In addition, other miRNAs were similarly expressed yet exhibited large differences in miRNA-mediated gene silencing [59]. Collectively, the level of a given miRNA is not a great predictor of silencing activity and the effect of upregulating a specific endogenous miRNA may not be readily apparent.

It was conceivable that access to the miRISC complex was limiting for gene silencing in our experiments. However, we found that the guide strand miRNAs were expressed at far higher levels than passenger strand miRNAs. This implies that the guide strand miRNAs were loaded into miRISC complexes because double stranded mature mRNA/miRNA* duplexes should give rise to equal expression of both strands. So despite loading of the p53-regulated miRNAs into the miRISC, there was no evidence for the downregulation of any targets tested. Recent evidence suggests that there are distinct populations of miRISC complexes [60] so that even the association of a given miRNA with miRISC is not a quantitative predictor of miRNA activity [59, 61]. High-throughput analysis indicates that most detectable endogenous miRNAs had no measurable effect on target expression and even highly expressed miRNAs can have minimal effects on target expression [61]. Therefore, our results support emerging information that suggests that miRNA expression in itself is not a strong predictor of miRNA activity.

Our work does not preclude a contribution of miR-34a-5p to p53-dependent cell cycle arrest but it suggests that the p53-dependent regulation of p21^WAF^ likely plays a greater role that miR-34a-5p. In support of this idea, Mir34 knockout mice, in which all Mir34 family genes were inactivated, retain a relatively normal p53 response and normal p53-dependent checkpoint function [62]. In contrast, p21 ^**WAF**^ knockout mice exhibit defects in p53-mediated G_1_ checkpoint function [63]. Our work suggests that other factors affect the efficiency with which specific miRNAs affect target mRNA and protein expression. These could include compartmentalization of these RNA populations, competition for binding with other miRNAs and/or RNA binding proteins or competition for binding due to the abundance of competing RNAs.

The HT29-tsp53 cell line used in the present work undergoes a sustained G_1_ arrest at the permissive temperature. Despite remarkable increases in the abundance of several p53-regulated miRNA and reports that p53 regulated miRNAs contribute to cell cycle arrest [15, 25, 51], there was no clear evidence of miRNA-mediated gene silencing against any of the targets tested at the mRNA or protein level, including key cell cycle regulatory proteins (Cdk4 and Cdk6) and oncogenes that indirectly regulate cell cycle progression (c-Myc and K-Ras). Instead, the G_1_ arrest was associated with increased expression of the cyclin-dependent kinase inhibitor p21^WAF1^. Therefore, the p53-dependent G_1_ arrest previously reported in these cells [31, 32, 37] appears to be independent of p53-dependent induction of miRNAs. Our results, in combination with mouse knockout studies, provide insight into the relative contribution of p53-induced miRNAs and mRNAs to the establishment of p53-mediated G_1_ and G2 arrests. Furthermore, our work serves as a caution to avoid over interpreting miRNA expression data alone.
